# A Review of Quality of Life Experienced by Patients Following Surgery for Pancreatic Cancer

**DOI:** 10.3390/cancers17162602

**Published:** 2025-08-08

**Authors:** Wendy Muircroft, Fraser Welsh

**Affiliations:** 1 Department of Palliative Care, Waikato Hospital, Hamilton 3240, New Zealand; 2 Department of Surgery, Waikato Hospital, Hamilton 3240, New Zealand; 3 Department of Surgery, University of Auckland, Auckland 1142, New Zealand

**Keywords:** pancreatic cancer, Whipples resection, quality of life, pancreatic insufficiency, cachexia

## Abstract

**Background**: There is evidence that pancreatic cancer arises from different pre-malignant conditions, which can take decades to transform to invasive cancers. Some individuals can benefit from surveillance and early access to surgery. Patients that have disease that is detected whilst it is resectable may have the disease cured by total or partial pancreatectomy. It is already known that pancreatic cancer, whether advanced or localised, causes impairment in quality of life, and survivors of surgery can have quality of life impaired by recognised and predictable pancreatic deficiencies. **Methods**: A literature review of key themes affecting quality of life in pancreatic cancer are presented here. **Results**: The biological features of the precursors of pancreatic cancer are described, with the rationale for surveillance and effects of surgical management in different conditions. **Conclusions**: Surgery for pancreatic cancer carries the potential for high impact on quality of life after survivorship after initial surgical intervention. Identification of issues that affect patients are discussed with the aim of improving the quality of care for patients.

## Simple Summary

Pancreatic cancer is relatively rare, and often presents when the cancer is advanced and is incurable. For patients that are suitable for surgery, which is the only curative option, there are risks of morbidity from a surgical procedure itself and significant impacts on quality of life arising from the effects of having either total or partial removal of the pancreas. Much attention in quality of life for patients surviving pancreatic surgery is focused on the impact of pancreatic enzyme insufficiency and pancreatogenic diabetes. The adverse impact of delayed gastric emptying also adversely affects quality of life. Other important quality of life concerns are the psychological burden of being diagnosed with cancer and undergoing surgery.

## 1. Background

Pancreatic cancer caused 3.3% of cancer cases diagnosed in the United States, but is responsible for around 8.4% of mortality due to cancer. Between the years 2015 and 2021 the average 5-year survival was 13.3% [1]. International studies have shown that the incidence of pancreatic cancer has increased each decade since 1990 on a worldwide scale. (See Table 1). The incidence of pancreatic cancer is expected to increase in the next decades. In one epidemiological study, the incidence was predicted to double for both men and women for the time period 2020–2044 [2]. The mortality from pancreatic cancer is due to the disease characteristics with non-specific symptoms that make it difficult to detect, and patients often present with non-curative (irresectable) disease at diagnosis.

Pancreatic cancer is a condition that can cause significant distress to patients and their carers, and can have an impact on the patient’s perception of quality of life in a number of different ways. Surgery is the only potentially curative treatment and is also undertaken to provide palliation for patients. Surgical procedures may give benefit to patients by providing symptom relief for the duration of the patient’s life.

## 2. Introduction

Common issues that affect patient quality of life after surgery were identified in 1998, and are still as applicable today as they were then [3]. These quality of life issues fall into the following broad categories:

- Post-operative complications, depending on the anatomy of the excision.
- Endocrine or exocrine insufficiency. These may lead to life-long requirement for dietary changes and supplemental medication.
- Impact on carers.
- Body image (this is related to cancer anorexia-cachexia syndrome primarily).

Improvements in morbidity and mortality after surgery have been made after decades of refinement of surgical techniques and case selection for the management of pancreatic cancer. The procedure that a patient may undergo depends on the tumour location and the histology.

## 3. Surgical Procedures and the Long-Term Sequelae

The surgical procedures that can be offered to a patient are determined by the histological subtype, the anatomical location of the tumour and the extent of disease elsewhere in the body. Oncological staging using TNM is not the only consideration in surgery for pancreatic cancer, where the operability of the cancer is a major consideration. Patients who do not have evidence of metastatic disease or evidence of vascular involvement would be considered ‘resectable’. Depending on the extent of vascular involvement (involvement of the superior mesenteric vein, portal vein, superior mesenteric artery or coeliac artery) patients may be considered ‘borderline resectable’ or ‘locally advanced inoperable.’ There is debate between clinicians regarding the definitions of ‘borderline’ but there is an international consensus that has produced a framework for patient assessment using radiological imaging to ensure consistency in patient assessment [4]. Patients with metastatic disease would not usually be offered surgery.

**Figure 1:**
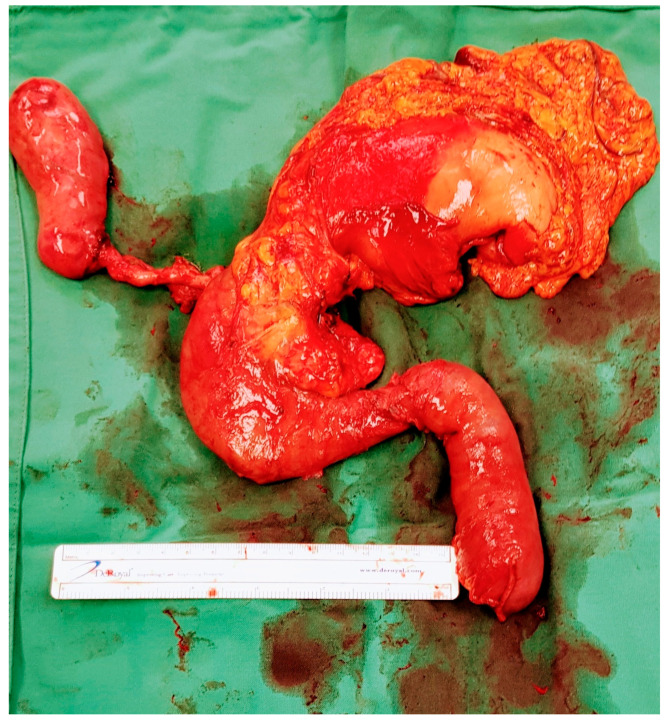
Whipple specimen.

There is a close relationship between type of surgery and outcomes. For tumours that are located in the head of the pancreas, Whipples resection is usually performed. This involves removal of the head of the pancreas, first part of the duodenum, gall bladder and bile duct. See Figure 1 In this procedure, the remainder of the digestive system is reconnected with the aim of restoring normal digestion. Pancreatic exocrine insufficiency commonly occurs after this procedure and is dependent upon the function of the remaining pancreatic tissue.

For tumours located elsewhere within the pancreas, either in the body or tail of the gland, partial pancreatectomy can be performed. Diabetes can be caused surgically in this instance. Surgery can lead to the complete removal of a tumour in the head or neck of the pancreas, and can improve survival and wellbeing. It can also expose the patient to risks which include post-operative complications, and digestive problems immediately after surgery. Some patients may require permanent dietary or lifestyle changes after surgery.

Total pancreatectomy is indicated when the primary tumour is unfavourably located in the centre of the pancreas, or when there are synchronous tumours. It results in Type 3 c diabetes and pancreatic exocrine insufficiency.

## 4. Tumour Biology and Histological Cancer Subtypes

Surgical management of pancreatic cancer is determined by several factors including operability, histology and anatomical location of the primary. Since the last century, it has been recognised that there are more than one type of pancreatic cancer, and the most common type, pancreatic ductal adenocarcinoma, can arise from malignant transformation of benign conditions. There are differences in therapeutics and outcomes for patients who are treated for primary pancreatic malignancies. The main histological significant subtype and associated precursor lesions are as follows:

- Pancreatic ductal adenocarcinoma (PDAC), the most common type of invasive cancer.
- Pancreatic intraepithelial neoplasia (PanIN).
- Intraductal papillary mucinous neoplasm (IPMN).
- Intraductal tubulopapillary neoplasm (ITPN).
- Mucinous cystic neoplasm (MCN).

Primary pancreatic neuroendocrine tumours (PNETs) can also arise from the pancreas and are also considered here.

### 4.1. Pancreatic Ductal Adenocarcinoma

**Figure 2:**
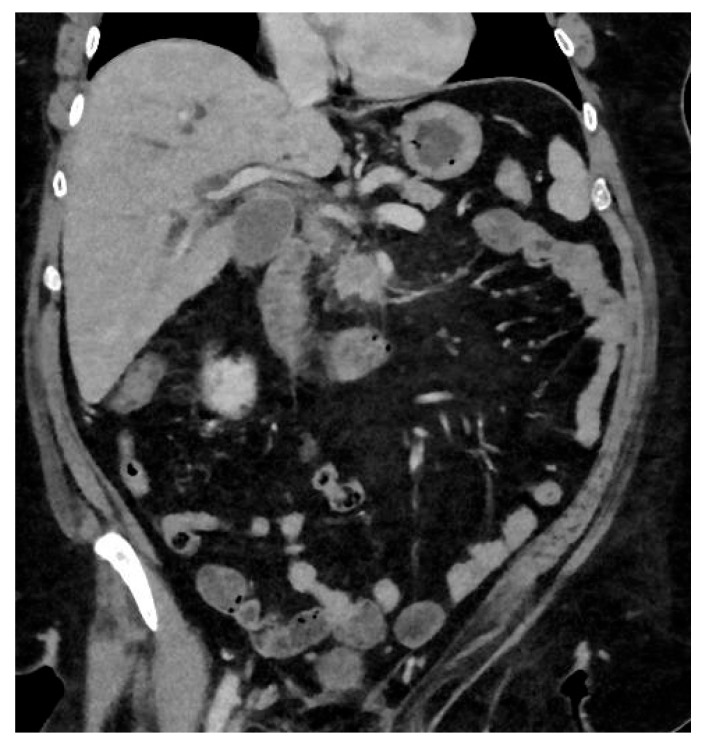
Hypodense lesion in the head of the pancreas, biopsy adenocarcinoma treated with Whipple.

Pancreatic ductal adenocarcinoma (PDAC) is an aggressive form of cancer that carries a poor prognosis. PDAC usually occurs in the head of the pancreas. It is estimated that between 80 and 85% of PDAC lesions are advanced and irresectable, or non-curable, at presentation. This is mainly because the symptoms that patients experience can be vague and non-specific, like abdominal pain, and the symptoms can be challenging to diagnose, leading to delays in diagnosis. See Figure 2.

Tumour markers that are often associated with pancreatic cancer such as CA19-9 and CEA, have poor sensitivity and specificity and are often not reliable in diagnosing pancreatic cancer, as some individuals with pancreatic cancer may not have any detectable elevation in these tumour markers. Currently there is no screening programme for pancreatic cancer, however there may be a genetic component to pancreatic cancer in up to 10% of cases and individuals with a familial history of pancreatic cancer can undergo interval screening in conjunction with genetic testing.

A combination of molecular studies and histological review of resected PDAC lesions have shown that it can occur concurrently with invasive and pre-malignant conditions leading to an established view that PDAC takes decades to transform from pre-malignant conditions.

### 4.2. Pancreatic Intra-Epithelial Neoplasia

Patients with a strong family history of pancreatic cancer can have multiple PanIN lesions which produce characteristic appearances that are similar radiologically to pancreatitis. There are common mutations associated with PanIN lesions and in broad terms these are categorised as tumour suppressor genes, oncogenes and genome maintenance genes (BRCA2).

The clinical significance of these findings are that patients with a genetic predisposition, e.g., BRCA1 or Lynch Syndrome, are at higher risk of developing pancreatic cancer arising from PanIN and there is a case for screening and long-term follow-up. Screening will be performed radiologically and will start from age 40 or 10 years younger than the age of their youngest affected relative. This is usually performed radiologically (usually by MRI) or endoscopically with endoscopic ultrasound [5].

### 4.3. Intra-Ductal Papillary Mucinous Neoplasm

**Figure 3:**
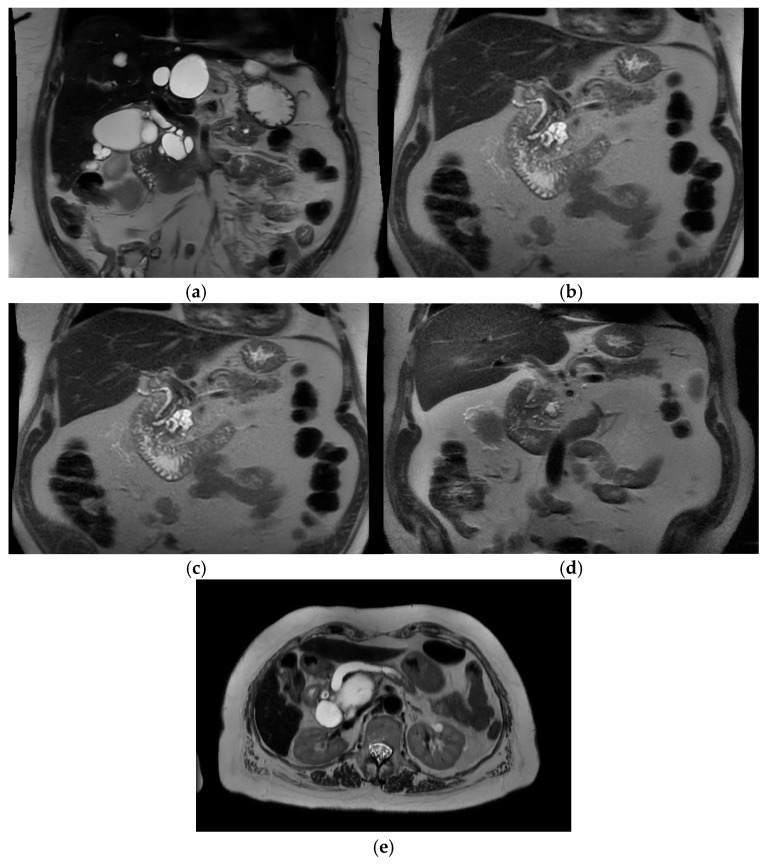
Branch duct IPMN and main duct IPMN. (**a**) Branch duct IPMN. Maximum size 3.2 cm. No other high risk or worrisome features. Incidental liver cysts. Resected—final histology IPMN with low-grade dysplasia. (T2-weighted MRI). (**b**) Branch duct IPMN >3 cm with thickened septations and nodularity—worrisome features. T2 Weighted MRI. Resected – final histology invasive cancer T3N2. (**c**) Same patient 1 year prior—approx. 3 cm size, “slightly thickened” septations. (**d**) Same patient 3 years prior. Smaller cystic lesion. No enhancing component. (**e**) Main duct IPMN—T2 weighted MRI showing grossly dilated main pancreatic duct.

Intra-ductal papillary mucinous neoplasm (IPMN) is a benign condition that has an evolving area of management in the realm of pancreatic surgery. IPMN can be an incidental finding on radiological examinations with CT of the upper abdomen, and may have management with initial surveillance before patients are considered for surgery. See Figure 3a–e.

**Figure 4:**
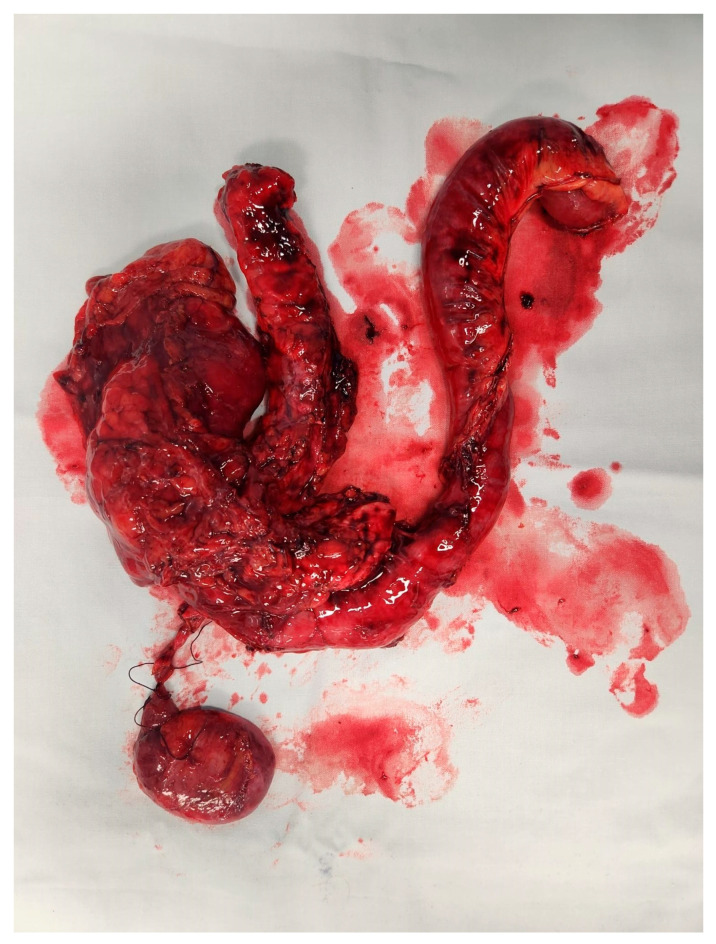
Total pancreatectomy for main duct IPMN.

The characteristic features of IPMN are papillary proliferation of mucin-producing epithelial cells which result in cysts arising from the pancreatic ducts. The cysts can block pancreatic ducts and cause pancreatitis, which is a painful condition that is associated with both IPMN and pancreatic cancer. IPMNs are divided morphologically into 2 groups, depending on whether it is located on the main pancreatic duct or on a branch duct. IPMNs can also be mixed type and show features of both main duct and branch type architecturally. See Figure 4.

The significance of IPMN from an oncological point of view arises from data derived from multiple observational studies which have confirmed that IPMN is a condition strongly associated with cancer. It can originate from several different cell lines within the body, with the intestinal and pancreaticobiliary subtypes having the worse outcomes. More specifically, IPMNs arising from the main pancreatic duct have a high incidence of ‘advanced histology, i.e., high grade dysplasia and invasive cancer. These IPMNs fit the morphology of high risk criteria. It was estimated that between 37 and 94% of resected specimens will show high-grade dysplasia and pancreatic cancer when they are reviewed by a pathologist [6].

Another study [7], estimated that 10–25% of IPMNs have concomitant pancreatic ductal adenocarcinoma (PDAC), based on information which has been gathered from patients who have undergone long-term surveillance after surgery.

Radiological criteria that indicate high risk features include: main pancreatic duct dilated more than 10mm, an enhancing solid component and biliary obstruction. Biliary obstruction only occurs where the IPMN lies within the head of the pancreas, in close proximity to the bile duct. Branch duct IPMNs tend to behave in a more indolent pattern, although they do carry a risk of malignant transformation over time, estimated to be around 15%. Surveillance is considered for patients who have branch duct IPMNs with a view to having pancreatic surgery while they are (a) medically fit for surgery and (b) do not show radiological features suggestive of malignant transformation at the outset [8].

Total pancreatectomy is increasingly being offered for IPMN when it is located in the main pancreatic duct. IPMN occurring in this area anatomically, are at risk of transforming to PDAC. The practice has evolved over time, and started to be reported approximately 10 years ago [9,10]. More detailed indications were outlined in 2018 [11].

The post-operative management of IPMN associated PDAC is similar in principle when PDAC has arisen de novo from the pancreatic ducts. Patients may have post-operative chemotherapy, but there was no consensus on the schedule that was administered in the ADENO-IPMN study [7]. It was reported that the overall 5 year survival for patients who had IPMN associated PDAC was 35%. In other studies, for comparison, the five year survival for patients who had surgical resection for PDAC arising independently from the pancreas, was as low as 11%. In the Panorama study [6], 3 year survival for pancreatic cancer was 18% and for IPMN surgery it was 84%. The ADENO-IPMN [7] study identified negative prognostic factors for survival and these were as follows: primary tumour location in the tail of the pancreas, increasing age, poor differentiation, increasing tumour size T3, lymphvascular invasion, perineural invasion, positive lymph node dissection N1 or N2, R Margin, and at least 1 major post-operative complication (Clavien-Dindo > 3).

### 4.4. Intraductal Tubulopapillary Neoplasm (ITPN)

ITPNs occur less frequently than other types of PDAC premalignant conditions. They are estimated to occur in <3% of pancreatic intraductal neoplasms. ITPNs differ from IPMN quite markedly in that they do not produce mucin and are not associated with cystic structures. However, ITPN also shows high grade dysplasia. Because it is a relatively rare lesion, there are few case survival series reported in the literature. However, the 5-year survival is estimated to be >30% [12].

### 4.5. Mucinous Cystic Neoplasm (MCN)

**Figure 5:**
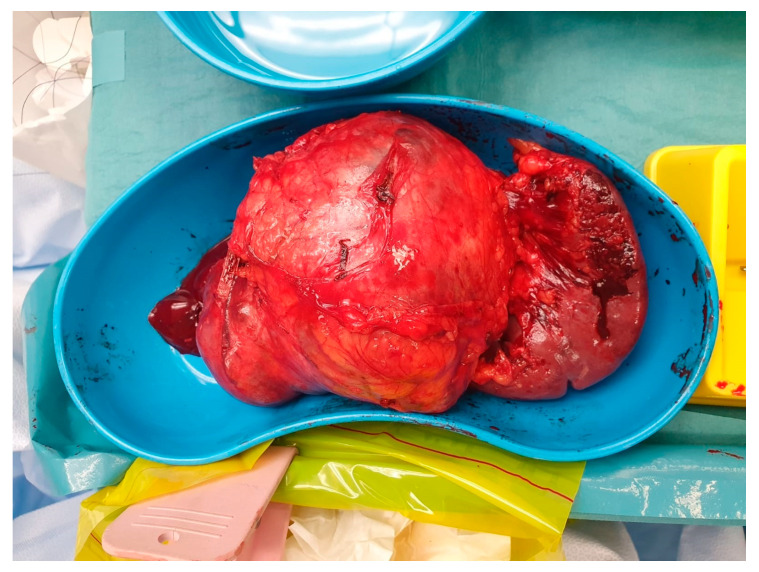
Distal pancreatectomy and splenectomy cyst.

MCNs are also premalignant conditions. They are more commonly found in the body or tail of the pancreas than in the head. See Figure 5. They show a female preponderance, with the incidence in over 90% of cases occurring in women. They are almost always unifocal, and complete resection is usually curative as there is only a very small risk of developing a metachronous tumour.

MCNs contain dense ovarian-type stroma that can express both oestrogen and progesterone receptors. Cyst fluid from MCNs can have elevated levels of the tumour markers CA19-9 and CEA, which can also circulate within the blood when there is invasive malignancy. The behaviour of MCNs is not reliable as invasive carcinoma can arise both from high grade and apparently benign appearing lesions [12].

### 4.6. Pancreatic Neuroendocrine Tumours

**Figure 6:**
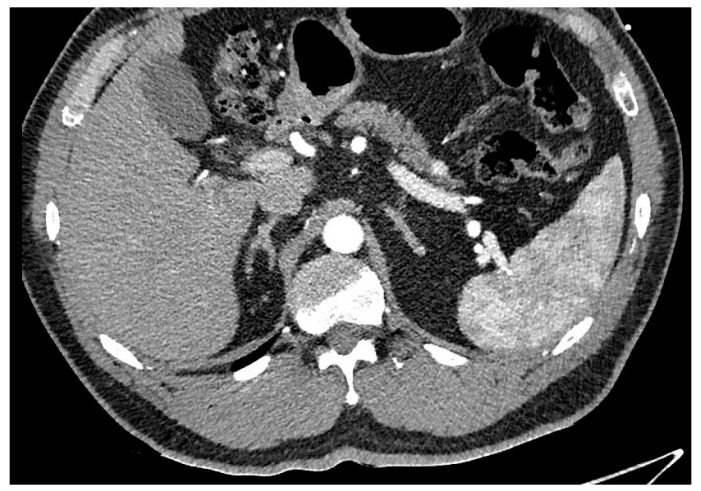
CT arterial phase with lesion in the tail of the pancreas consistent with neuroendocrine tumour.

**Figure 7:**
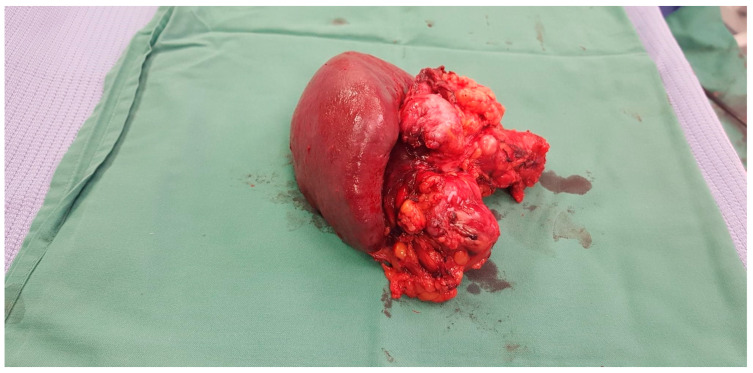
Distal pancreatectomy and spleen NET.

Pancreatic neuroendocrine tumours (PaNET) are classified into 2 broad groups: hormone-secreting (functional) or non-secreting (non-functional) tumours. Insulinomas and gastrinomas are the most common types of functioning tumours. Non-functioning tumours that are smaller than 2 cm are usually managed with surveillance until they measure more than 2 cm, which is regarded as a critical size. See Figure 6. Depending on the anatomical location of the primary, they can be managed with Whipples resection, distal or total pancreatectomy. See Figure 7 Functional tumours smaller than 1 cm may be treated with enucleation of the tumour, leaving as much as possible of the pancreatic parenchyma intact.

Multiple endocrine neoplasia type-1 (MEN type 1) is a relatively rare subtype of primary pancreatic cancer. It occurs more frequently in younger patients than older patients, and is often multi-focal in origin. There are no current guidelines for the surgical management of MEN Type 1 PaNET. For this reason it is often managed by total pancreatectomy. When survival for patients who had sporadic (non-hereditary) endocrine neoplasia was compared with the MEN type 1 population who had surgery, both groups had equivalent survival [13].

## 5. Surgical Procedures—The Indications and Impact on Quality of Life

Primary pancreatic ductal adenocarcinoma at the head of the pancreas is the most commonly occurring pathology. Both Whipples procedure (PD) and pylorus preserving pancreaticoduodenectomy (PPPD) are performed, with Whipples being the most common. Other procedures discussed here have not necessarily universally been adopted as practice in all upper gastrointestinal surgical units. There is variability in local expertise based on training and practical influences. Differences in quality of life after undergoing the respective procedures are observed. PPPD is better for quality of life. In the Cochrane review [14], in the post-operative period, there was no difference in between the 2 groups for morbidity but there were improvements in weight, exocrine insufficiency, and quality of life after PPPD.

The authors found that there was a statistically significant improvement in weight and quality of life in which favoured patients who were treated with PPPD. In another study, the authors reported that, following PPPD surgery, the thickness in millimetres of the pancreatic remnant was a predictive factor for the presence of PEI [15].

With careful patient selection, patients may be offered total pancreatectomy if there is a soft pancreas or compromised pancreatic duct, multi-focal ductal disease, or considered in congenital pancreatitis. In this rare condition, patients may also be offered islet cell implant. Congenital pancreatitis is regarded is an autosomal dominant condition, which results in multiple episodes of pancreatitis that can progress to chronic pancreatitis. Congenital pancreatitis is a pre-malignant condition, with an estimated 40% risk of transformation to pancreatic cancer [16].

Renal cell carcinoma can metastasise to the pancreas. Autopsy data confirms that 2% of renal cell cancers have pancreatic metastases at their time of death. Typically, renal cell carcinoma metastases within the pancreas occur several years after successful treatment of a primary renal cell cancer, and can be a solitary metastasis within the pancreas. Where there is oligometastatic disease with no metastases elsewhere, partial resection can be offered [17]. See Table 2 for a summary of the indications for surgical procedures.

### 5.1. Immediate Post-Operative Complications

The International Study Group for Pancreatic Surgery (ISGPS) has standardised the definitions of the most common complications of pancreatic surgery, and these are pancreatic fistula [18], post-pancreatectomy haemorrhage, delayed gastric emptying [19] and post-pancreatectomy bile leak. This includes stratification of the severity of each complication. These definitions are useful as tools for clinical audit, research and comparison of outcomes between units.

### 5.2. Post-Operative Pancreatic Fistula and Post-Operative Haemorrhage

Post-operative pancreatic fistula (POPF) is thought to arise when there is failure of new tissue growth in the pancreatic anastomosis. This causes a leak of pancreatic juice and enzymes into the abdomen. It results in longer hospital admission. The most significant morbidity are related to persistent drainage (longer than 3 weeks), signs of infection, sepsis. Some of these require readmission to hospital and in the most severe cases, can cause death [20].

Postpancreatectomy haemorrhage (PPH) is usually a sequelae of POPF, as erosion of the blood vessels in close proximity to the fistula can occur [21]. The ideal management of haemorrhage under these circumstances is with the use of diagnostic radiology and intervention, usually endovascular embolization, if there is an active bleeding site. Re-exploration of the abdomen post-operatively has a high association of complications and morbidity. The immediate impacts for haemorrhage are delayed recovery, in-hospital complications and prolonged hospital stay. Treatment for a haemorrhage (as with other complications from surgery) can affect future care with chemotherapy either by causing treatment delay or permanent deferment due to deterioration in performance status whilst patients are in hospital.

### 5.3. Delayed Gastric Emptying

Delayed Gastric Emptying (DGE) is a post-operative phenomenon that is reported to occur in up to 30% of patients after pancreaticoduodenectomy. It occurs when the normal physiological process of the stomach emptying into the small intestine is disturbed. The patient can report the following symptoms: feeling of abdominal fullness, nausea and vomiting and inability to establish regular diet. It is a source of frustration to both patients and clinicians, as it leads to prolonged hospital admission after surgery, and there are few effective therapies to treat the condition. Often short-term management can involve the use of pro-kinetic medication or the insertion of a naso-gastric tube, with variable degrees of success. There are financial implications that arise as well for patients with the prolonged hospital stay.

It is a condition that is reported to be associated with adverse outcomes following surgery due to impaired access to chemotherapy and radiotherapy as a result of relatively poor nutrition status compared with patients who do not experience DGE [22]. The immediate post-operative complications often improve as a result of treatment, and the long-term impact of pancreatic surgery for pancreatic cancer will be considered separately.

## 6. Quality of Life Assessment Tools—Data from Patients That Have Undergone Total Pancreatectomy

It has been recognised for decades that there are certain features of surgery that can impair patients’ perception of quality of life. See Table 3 for a summary. The EORTC QLQ 30 is used as a tool for assessment of quality of life in cancer patients, and it was recognised that patients with pancreatic cancer have specific needs so the EORTC QLQ PAN 26 was designed for application with the EORTC QLQ 30 [3]. This was initially planned as a research tool to obtain cross-sectional data on the patient’s experience of the condition and its treatment, with changes in each patient reported over time. It is also helpful as a tool to focus on further treatment or investigations for each of the domains that have a high score.

The PANORAMA study [6] was a nation-wide multi-centred study that collated results from different centres and is probably the biggest study to date to report on outcomes on quality of life after total pancreatectomy compared with the general population. The Panorama study [6] validated the use of EORTC QLQ 30 in combination with EORTC QLQ PAN 26. The authors reported that: ‘Compared with the general population, patients who had a total pancreatectomy had more financial difficulties, diarrhoea, appetite loss, insomnia, dyspnoea and fatigue.’ Patients also reported worse global health status compared with the normal population that were sampled.

A new tool to evaluate the sequelae of pancreatic exocrine insufficiency was published in 2019 [23]. The tool measured 26 items relating to quality of life and was designed to have more specific evaluation of the patient’s reported experience with PEI. Its use and efficacy has been evaluated in the above study published by the authors. However, in this study the patient population who were recruited were diagnosed with chronic pancreatitis or cystic fibrosis. In the future, the use of the tool could be extended to patients with pancreatic cancer.

Patients with pancreatic cancer have poorer quality of life scores than other patients who are diagnosed with cancer [24]. They report somatization of stress when compared with other cancer patients. Dysregulation of the immune and endocrine systems occur when patients report chronic stress. They can struggle with fear of recurrence of the disease and poor prognosis. Patients with poor psychosocial support have poorer outcomes in terms of greater incidence of anxiety, depression and physical symptoms than patients who have good support strategies, with the highest levels of satisfaction expressed by patients who have good family support [23].

The most common complications arising from pancreatic surgery are diabetes and pancreatic exocrine insufficiency and these can have a significant impact on quality of life and sense of wellbeing.

## 7. Long-Term Endocrine and Exocrine Complications of Pancreatic Surgery

Both diabetes and pancreatic exocrine insufficiency require subspecialty care for ongoing monitoring and treatment, and can result in significant weight loss. These conditions are considered in turn.

### 7.1. Pancreatogenic (Type 3c) Diabetes

Pancreatogenic diabetes is often regarded as the most significant factor that influences quality of life either after partial pancreatic resection or total pancreatectomy. It is related to the consequences of insulin deficiency and the effects of this on the liver and the peripheral organs as there is a lowering of endogenous hormone levels. After pancreatic surgery, in addition to insulin deficiency, there is a new metabolic state which arises, where there is increased peripheral sensitivity to insulin and a relative deficiency in glucagon due to loss of alpha cells. This is why hypoglycaemia is common in pancreatogenic diabetes, which is different to other forms of diabetes where either insulin deficiency or insulin resistance lead to hyperglycaemia. This phenomenon has led to it being described as ‘brittle diabetes’ [25]. Hypoglycaemia occasionally has more severe ramifications, leading to a hospital admission, permanent damage to the central nervous system, or even death.

There was a difference reported in the psychosocial distress associated with diabetes with patients who had a high risk pancreaticoduodenectomy and those who had a completion pancreatectomy for pancreatic cancer. The psychosocial impact of diabetes and the need for insulin therapy were greater after completion pancreatectomy than high risk pancreaticoduodenectomy [26]. In this study, the psychological burden of having diabetes was reported as being more significant in the those who underwent completion pancreatectomy.

There have been improvements made in the management of blood sugar levels with the advent of insulin continuous infusion and monitoring devices. The PANORAMA [6] study has reported that most recent studies are finding that there has been a significant improvement in the management of Type 3c DM. There was no difference in distress perceived by total pancreatectomy patients who have treatment for diabetes than the group of patients who are diagnosed with Type 1 diabetes. Enhanced satisfaction with care observed by patients who have treatment with a continuous subcutaneous infusion of insulin compared with patients who have multiple daily injections. Patients who have a TP for the indication of IPMN are not eligible candidates for islet cell auto-transplantation, so they resort to using insulin infusions or injections. The islet cell transplantation is commonly not available in the Netherlands.

It is recommended for patients to have follow up with a diabetic endocrine specialist team, and the results are better if the endocrinologist has more experience in the care of patients that have had a total pancreatectomy. For ongoing support, patients require education on how to manage diabetes monitoring and medication administration, and will require follow-up in outpatient clinics from an endocrinologist, a dietician and diabetic specialist nurse.

Type 3c diabetes mellitus management needs a collaborative approach, with the patient having new responsibilities in independently managing the long-term consequences of different areas of patient functioning. These include: self-injection, monitoring of glucose and ketones, weight management and dietary intake, exercise, awareness of the legal implications of diabetes on driving, employment, travel and alcohol intake [27].

In the PANORAMA study [6], pre-operative diabetes (NOD) was present in 60 or 41% of patients. Following total pancreatectomy, new-onset diabetes was present in 87 patients or 57%. The study also reports that one patient had an autologous pancreatic islet cell transplant, and this was successful as the patient did not develop new-onset diabetes.

Also reported in the PANORAMA [6] study was the significant risk of hypoglycaemia. One patient died of hypoglycaemia post-operatively. It was not known whether it was a factor contributing to the deaths of other patients as there was insufficient data. Insulin pumps with continuous glucose monitoring could theoretically lead to a reduced risk of post-operative hypoglycaemic episodes.

However, participants in the DIAMOND study [28] reported satisfaction with the device which had continuous subcutaneous infusion of insulin and continuous glucose monitoring compared with controls who had intermittent glucose injections. This did not translate into an overall reduction in hypoglycaemic worry. The subjects that had the continuous glucose monitoring and insulin infusion also reported feeling better overall but it did not follow on to feelings of satisfaction with better parameters of glycaemic control.

### 7.2. Pancreatic Exocrine Insufficiency

Pancreatic exocrine insufficiency (PEI) is known to impair quality of life [29]. PEI can occur in multiple conditions affecting the pancreas, but it commonly occurs in pancreatic cancer, either caused the cancer itself, or arises as a permanent consequence of surgery to the pancreas. PEI can cause several problems such as poor nutrition, delayed growth, increased infection rates (in Cystic fibrosis) and increase the incidence of cardiovascular events.

PEI is established when only 56–60% of dietary fats are absorbed and causes fat malabsorption due to the reduced release of enzymes that digest dietary fats, such as lipase and trypsin. It occurs with pathological conditions of the affecting the head of the pancreas, as the area of production of lipases is concentrated within the head of the pancreas. Hence, if the main pancreatic duct is blocked due to cancer, for instance, PEI will occur. Also, if the head of the pancreas is excised, it is very likely that the patient will have PEI in the post-operative period. Surgery that only involves partial excision of the more distal area of the pancreas, such as resection of the tail or body of the pancreas, is much less likely to cause PEI. PEI can be closely related to the residual volume of pancreatic tissue after surgery, and also depend on the anatomical location of the residual tissue.

### 7.3. Evaluation of Pancreatic Exocrine Insufficiency

The incidence of PEI is known to be underdiagnosed [23]. Currently there is no consensus on the assessment and therapeutic monitoring of PEI. It can be diagnosed with tests such as faecal fat analysis, C13 breath testing with a mixed triglyceride drink, faecal elastase or pancreatic function test (not currently applied in clinical practice as it is an invasive test) [30]. When PEI is confirmed, patients can commence pancreatic enzyme replacement therapy (PERT).

### 7.4. Pancreatic Enzyme Replacement Therapy

The principle of PERT is to replace the patient’s endogenous pancreatic enzymes with porcine pancreatic extract which is prepared as a capsule which contains microspheres of pancreatic extract. The capsule is taken by mouth and is pH sensitive, therefore the optimal time for patients to take the capsule is whilst they are eating a meal or snack. Often, at-risk patients may not be screened for PEI, or receive a prescription for PERT. In the PANORAMA [6] study, enzyme supplementation was prescribed for 16% of pre-operative patients and increased to 88% before the patients were discharged. The incidence of PEI or diagnostic testing is not quoted in this study.

Patients need to be given information about the use of PERT. 25,000 IU of lipase per meal and 10,000 IU per snack are recommended starting points for the dose of PERT. There is a significant role for dieticians as patients require education about how to take PERT, calculate the fat content of a meal or snack, and need ongoing monitoring to assess their nutritional status. Micronutrient deficiencies are common with PEI [31]. There are other preparations of PERT available in the United States and these include Viokace, a non-enteric coated tablet, and Relizorb an in-line lipase cartridge, that can be added to enteral feeding formulae [32].

In the United Kingdom, Pancrex (pancreatin) powder is available. It is used by mixing with water and flushing in an enteral feeding tube or can be added to a feed in a feeding reservoir. The dose of this can be titrated [33]. For patients who may have either religious or philosophical objection to the use of a porcine extract medication, there are other supplements available over the counter. However, these do not have equivalent properties to the ones described above, and patients should be specifically counselled about this.

Where treatment is unsuccessful it is important to check patient compliance, and the dose of enzymes required. For patients who fail to respond to therapy, it is important to check for other causes. The differential list of post-operative causes include: Blind loop syndrome resulting in bacterial overgrowth, post-cybal asynchrony (where there is a mismatch between the release of pancreatic enzymes and the arrival of digested food or chyme in the duodenum), and dumping syndrome. Dumping syndrome occurs when the pylorus of the stomach prematurely releases only partially digested food into the small bowel, causing a cascade of internal events where there is a mismatch with hormones, blood sugar and fluid volumes within the body. Specific treatments are required to address these specific complications. For guidelines on the management of PEI see [31].

### 7.5. Cachexia and Sarcopenia

Cancer anorexia-cachexia syndrome (CACS) is multi-factorial in aetiology. Cachexia is often mediated by inflammatory cytokines that are released systemically. It is frequently complicated by nausea and vomiting, reduced oral intake, PEI and diarrhoea due to other causes. Unintentional weight loss that occurs in pancreatic cancer can be catastrophic for these reasons and results in impairment of body image and wellbeing, as well as being a source of distress to carers [29].

At the present time, there are no consensus guidelines for the pre-operative assessment and management of preoperative cachexia in pancreatic cancer [34].

### 7.6. Preoperative Sarcopenia

Sarcopenia is a relatively common finding in patients who are diagnosed with pancreatic cancer. One of the means of assessing it is to score the lean muscle mass in the psoas muscle at L3 on CT scan imaging [35]. This systematic review compared the outcomes for patients who had sarcopenia compared with patients who were designated to have no sarcopenia. Overall the authors found that patients who had sarcopenia were more likely to have a prolonged hospital stay following pancreatic resection. It was not reflected in an actual increase in complications from surgery, and there was no reported increase in morbidity and mortality. There have not been any studies that look at quality of life in patients who are clinically diagnosed with sarcopenia.

These findings were in contrast where there was a strong association between preoperative muscle wasting and worse post-operative outcomes. Sarcopenia was also a significant risk factor for post-operative pancreatic fistula, increased length of hospital stay and patient discharge to a residential aged care facility. Sarcopenia was also associated with poor tolerance of adjuvant chemotherapy and was a predictor of earlier recurrence of disease.

### 7.7. Psychosocial Impact of PEI for Patients with Pancreatic Cancer

The experience of PEI can be significant for patients as the weight loss can have devastating impact on weight, cancer cachexia and body image. If it is untreated, PEI can cause significant restrictions to daily life. In severe cases, patients can experience frequent, often uncontrollable bowel motions, with offensive smelling diarrhoea and abdominal pain. 26 points or issues associated with PEI were assessed in a paper that validated the tool in the patient population who have cystic fibrosis or chronic pancreatitis [23].

PEI can lead to social isolation, and make patients feel unwilling to leave their homes, and they may significantly reduce social visits and outings. Some patients report that they need to plan activities based around the location of toilets, so-called “toilet dependence” [29].

PEI also has significant impact upon carers. Managing diet can be a source of tension and conflict between patients and carers. The origin of this is often a lack of appetite and food refusal after a meal has been prepared by carers. Carers often reported a high sense of distress due to their inability to successfully manage diet and prevent weight loss. They also reported distress about a variety of issues, which included the co-ordination of health care provided to their loved one, lack of education about PERT, inability to access PERT at the appropriate time, and inability to access diabetic-appropriate nutritional supplements.

PEI often has a negative impact on wellbeing after surgery for pancreatic cancer. However, there are systemic features of pancreatic cancer that are associated with depression, which has its own effect on wellbeing.

## 8. Depression and Pancreatic Cancer

Depression is known to have a high incidence in pancreatic cancer, the published estimates vary as much as 41–71% [36]. The incidence of depression is high when patients are diagnosed with pancreatic cancer, and it is reported to be higher than other upper gastrointestinal cancers. It has such a strong historical association with pancreatic cancer that it used to be considered to be a psychological presenting symptom of depression [37]. Patients with pancreatic cancer also report symptoms of higher levels of anxiety than the general population.

The aetiology of depression in pancreatic cancer has not been definitively elucidated. However, there are several active hypotheses for the mechanism of depression in pancreatic cancer, some of which have a theoretical basis for pharmacological management of the condition [38].

Pancreatic cancer is associated with increased levels of inflammatory cytokines such as IL-6, increased urinary excretion of 5-HIAA, 5-HT and increased metabolism of serotonin, formation of antibodies to serotonin and production of neuropeptides. NICE guidelines from the United Kingdom recommend the use of serotonin-selective reuptake inhibitors for depression associated with cancer, and with multi-factorial causes of serotonin depletion, this class of anti-depressants may have some efficacy to treat depression in pancreatic cancer. With the high prevalence of anxiety in patients with pancreatic cancer, either benzodiazepines that have primarily anxiolytic properties or an antidepressant with dual therapeutic role to treat depression and anxiety, such as mirtazapine can be considered. Using the minimal dose of benzodiazepines possible is preferred as they carry a risk of causing falls and are associated with psychomotor retardation and fatigue which is often a problem in pancreatic cancer.

Neuropathic pain due to coeliac plexus nerve infiltration commonly occurs with pancreatic cancer. Tricyclic antidepressants such as amitriptyline or nortriptyline with the more favourable anti-cholinergic side effect profile, were traditionally used as first line agents in this setting. However, they can still be considered to treat neuropathic pain in lower doses but they have been superseded by the use of gabapentinoids such as gabapentin or pregabalin for this indication. Traditional tricyclic antidepressants can also be contraindicated with cardiac conditions, as well as causing dry mouth and constipation which some patients can find intolerable. There is ongoing research into the role of hallucinogenic medications such as ketamine and psilocybin in the management of depression. Whilst they are being used more commonly to manage conventional antidepressant-refractory depression, their role in the management of depression due to pancreatic cancer is not established at the present time.

For treatment of refractory cases of depression associated with pancreatic cancer, a referral to a local psychiatry service can be helpful. As well as antidepressants, patients can also benefit from access to cognitive behavioural therapy and other specialised therapies. The presence of depression can lead some patients to fail to engage with supportive care services or comply with medication and as a consequence, depression can have an adverse effect on wellbeing and quality of life. Patients can be screened for the presence of anxiety and depression using a well-established tool like the well-validated Hospital Anxiety and Depression Scale (HADS). Some specialist palliative or supportive care services in different regions have access to liaison psychiatry services and provide the routine screening of patients as part of the multi-disciplinary management of pancreatic cancer.

## 9. Palliative Care in Advanced Pancreatic Cancer

Patients with advanced pancreatic cancer can benefit from an early referral to a palliative care service to help improve comfort and wellbeing. In addition to metabolic complications arising from surgery described above, they commonly experience symptoms such as pain and nausea and vomiting. They may also experience complications from the disease itself.

Patients who have relatively less pain with pancreatic cancer often have better reports of quality of life. Pain can cause disturbed sleep, reduced eating and calorie intake, and professional and social isolation. Pain due to pancreatic cancer can be multi-factorial in aetiology, and accurate diagnosis is essential for successful management. Pain is often neuropathic in nature when there is infiltration of the nerves of the coeliac plexus and may require multi-modal treatment with a combination of opioid and adjuvant analgesics. Treatment failure can occur with these agents. It is not known whether a conditions such as PEI or changes in gastrointestinal motility caused by surgery or opioids can alter the absorption profile of oral analgesic agents.

For pain that is complex by virtue of being refractory to oral opioids, or poorly tolerated, a multi-disciplinary approach is required and often a referral to an interventional pain specialist can help with pain management. Coeliac plexus neurolysis is the gold standard treatment and can be given via different routes, depending on local expertise. Some centres practice prophylactic coeliac plexus block either intra-operatively at the time of definitive surgery, or at the time of biopsy, often performed via endoscopic ultrasound scan. Interventional radiologists may perform it under direct imaging, and there are multiple approaches to this. Some centres have access to splanchnicectomy, where there is surgical removal of the ventral sympathetic ganglia.

Tumours originating in the body and tail of the pancreas can over-express tissue factor or mucin. Both of these are responsible for an increased risk of venous thrombo-embolic disease (VTE) compared with tumours that are located within the head of the pancreas. Where patients present with abdominal pain, inferior vena cava or splanchnic vein thrombosis should be considered in the differential list of causes. There is currently no evidence to support the routine use of thromboprophylaxis in pancreatic cancer.

Liver failure and jaundice are common features when patients present with advanced pancreatic cancer. Biliary stents can be complicated by ascending cholangitis and often requires treatment with intravenous antibiotics. If colonisation is suspected clinically, replacement of the stent can be considered. Pruritis often occurs with jaundice, and some of the recommended treatments like cholestyramine or rifampicin are often poorly tolerated or unsuccessful and further research is required [39].

## 10. Future Directions

Pancreatic cancer has well-established precursor lesions, which, if identified and managed early on leads to surgery for pancreatic conditions with potentially better oncological prognosis. The increased use of cross-sectional imaging means more patients may be diagnosed with early lesions such as IPMNs that have adverse radiological features but are not yet invasive cancer. Similarly NETs although relatively rare, may be detected earlier when they are small neoplasms. For this group of patients with better prognosis lesions, quality of life after pancreatic surgery will become an increasingly important issue. Likewise, improvements in perioperative outcomes such as declining early mortality, means that there are more patients for whom quality of survivorship rather than mere survivorship matters.

Increasing use of neoadjuvant approaches to PDAC may change the profile of patients having surgery for invasive cancer in two ways: (1) Downstaging tumours pre-operatively may allow more patients to have surgery with curative intent and mean that more patients complete systemic treatment as well as surgery, which leads to longer survival. (2) Patients with aggressive lesions may progress while undergoing neoadjuvant treatment and this group may be spared high impact surgery that would have been futile, although the final outcome is palliative.

PDAC is associated with poor prognosis, which is often attributed to the multifactorial nature by which it evolves. It is difficult to treat successfully with systemic therapy as it often develops resistance to treatments due to frequent mutations and demonstrates genetic heterogeneity within the same primary tumour. Treatments that have been successful in targeting single genes in other types of cancer such as EGFR in lung cancer or B-RAF in melanoma, are not effective in pancreatic cancer for this reason [40].

Standardised measures of quality of life after pancreatic surgery such as EORTC QLQ Pan 26 [3] and PEI Q [23]. The PEI Q can be used to identify areas of care that warrant further research. Improvements in type 3c Diabetes management and its monitoring can potentially improve quality of life in this patient group, potentially making total pancreatectomy, where indicated, a feasible option for more patients. Early involvement of diabetic specialist teams for patients who have undergone pancreatic resection is also important.

Increased recognition of the importance of PERT can lead to better outcomes where this has been neglected historically. Involvement of multidisciplinary teams including dieticians and credentialled clinical nurse specialists may reap benefits. Involvement of palliative care physicians to assist with symptom control where appropriate is likely to be advantageous. There is a role for early referral of patients to supportive or palliative care due to the high burden of symptoms and poor prognosis. Interventions such as coeliac plexus neurolysis should be considered early when appropriate. The impact of delayed gastric emptying and other digestive disturbances after pancreaticoduodenectomy has tended to be under-recognised and resolving this issue remains a challenge. Further research into this problem, its solutions and its impact may be important for improving quality of life for pancreatectomy survivors.

## 11. Conclusions

Surgery for pancreatic cancer carries a potential for high impact on quality of life after survivorship of initial surgical intervention While attention is rightly focused on perioperative morbidity and mortality the sequelae of surgical complications, endocrine and nutritional implications of pancreatic surgery have far reaching consequences both for patients and their carers. Assessment tools for quality of life have been developed and may be useful both from an investigative perspective and as an adjunct for professionals involved in post-surgical care of pancreatic cancer patients DGE and its impact on quality of life is likely under-recognised and worthy of further research. Valid concerns about the endocrine impact of total pancreatectomy (diabetes related QoL) have limited the use of total pancreatectomy but improvements in management of Type 3c diabetes may make this intervention more feasible for more patients.

## Figures and Tables

**Table 1:** Published data on the international incidence and mortality due to pancreatic cancer.

Study	Incidence	Mortality
*International PatternsIn Incidence And Mortality Trends Of Pancreatic Cancer In The Last 3 Decades: A Joinpoint Regression Analysis Ilic I and Ilic M World Journal of Gastroenterology 2022 Vol 28 Issue 28 p4698*–*4715* *doi: 10.3748/wig.v28.i32.4698*	There were 495,773 new cases worldwide reported in 2020; 26,2865 were males and 232,908 were females.	A total of 466,003 deaths from pancreatic cancer were reported worldwide in 2020. Increased incidence in males 246,840 compared with 219,163 females
	The highest incidence in new cases were reported in the Western Pacific Region, where there were 182,074 new cases or 39.1% in this area.	
*Global, Regional, and National Burden Of Pancreatic Cancer From 1990 to 2021, its attributable risk factors, and projections to 2050: A systematic Analysis Of The Global Burden of Disease Study 2021 Li T. et al BMC Cancer 2025 Vol 25 Issue 189 p1-26* *doi: 10.1186/s12885-025-13597-z*	Global incidence has increased significantly between 1990 and 2021; in 1990, the total number of cases was 207,905; in 2021, there were 508,533 new cases.	Highest mortality noted in 70–74 years age group. Mortality by region by descending incidence: Central Europe, High-income Asia Pacific, Eastern Europe, High-income North America, Western Europe (top 5 by highest mortality). Increased incidence in males in these groups.
	Highest age standardised incidence rates (ASIR) in 2021 were recorded in high-income Asia Pacific (10.69), which was followed by high-income North America (10.2).	
	*Key points—pancreatic cancer and mortality are increasing in incidence and mortality worldwide. There is a greater incidence in males than females and peak in 70–74 years*	

**Table 2:** Surgery and quality of life.

*Anatomical* *Location and Procedure*	*Complications*	*Impact on Quality of Life*
*Head of the pancreas, usually Whipples Resection or Pylorus Preserving Pancreatico-Duodenectomy (PPPD)*	*Loss of Pancreatic Exocrine Function*	*Taking Pancreatic Exocrine Replacement Therapy* *Permanent Dietary and lifestyle changes*
	*Delayed Gastric Emptying more Common with Whipples Procedure*	*Taking medication to promote gastric motility, sometimes prolonged hospital admission in the post-operative period*
*Solitary tumours located in the body or tail of the pancreas managed with distal Pancreatectomy*	*Loss of islet cells which produce insulin and loss of alpha cells in the Islets of Langerhans which produce Glucagon*	Causes Diabetes Permanent lifestyle and dietary changes with taking medication Anxiety about hypoglycaemia
*Multi-focal tumours, large IPMNs Carefully selected patients undergo Total pancreatectomy*	*Loss of islet cells and alpha cells*	*Causes Type 3c Diabetes Permanent lifestyle and dietary changes Taking medication, monitoring blood sugar levels anxiety about glycaemic control, brittle diabetes*
	*Loss of pancreatic exocrine function*	*Taking pancreatic exocrine replacement Therapy Permanent Dietary and lifestyle changes*
	*Key messages: Surgery for pancreatic cancer often requires lifelong dietary and lifestyle changes, due to specific endocrine or exocrine deficiencies*	

**Table 3:** Quality of life issues after surgery for pancreatic cancer.

*Issue Category*	*Health Issues and Management*	*Impact on Quality of Life*
*Post-operative complications* *See Table 2 for details*		
*Endocrine insufficiency*	*Type 3c Diabetes, rare form of diabetes control can be difficult to achieve due to loss of glucagon.*	*Permanent glucose monitoring Automated devices available in some countries*
		*Anxiety about diabetes monitoring*
		*Cost and accessability of medication, education, compliance*
		*Permanent dietary and lifestyle changes*
	*Islet cell transplant available in some countries*	*Accessibility issues*
*Exocrine Insufficiency*	*Pancreatic exocrine insufficiency (PEI) Leads to steatorrhoea, weight loss, cachexia syndrome, micronutrient deficiencies*	*Permanent medication with pancreatic enzyme replacement therapy.* *Toilet dependence, anxiety about faecal incontinence*
		*education, compliance*
*Impact Upon carers*	*Concerns about diet and weight loss*	*Relationship stress due to conflict about meals and eating*
	*Financial toxicity from treatment; toxicity from treatment*	*Anxiety and stress, loss of home*
	*Employment breaks/loss of employment*	*Financial stress*
*Body Image*	*Weight loss due to multifactorial causes such as PEI, permanent changes to gut function following surgery, cancer anorexia-cachexia syndrome*	*Anxiety and distress due to weight loss, which can be catastrophic*
		*relationship disruption, changes in sexuality, loss of employment role*
*Psychosocial factors*	*Depression and anxiety often coexist in pancreatic cancer*	*Timely and accurate diagnosis, Commencing medication and access to psychiatry services if required*
	*Financial stress*	*Access to social work support*
	*Concerns about cancer recurrence*	*Access to specialty cancer psychology services*
	*Concerns about death/dying/physical symptoms*	*Access to Palliative care*
	*Key messages—pancreatic cancer surgery affects quality of life in multiple different domains.*	
